# Polymer-brush-afforded SPIO Nanoparticles Show a Unique Biodistribution and MR Imaging Contrast in Mouse Organs

**DOI:** 10.2463/mrms.mp.2016-0067

**Published:** 2017-01-30

**Authors:** Ting Chen, Yuki Mori, Chizuko Inui-Yamamoto, Yutaka Komai, Yoshiyuki Tago, Shinichi Yoshida, Yoshitsugu Takabatake, Yoshitaka Isaka, Kohji Ohno, Yoshichika Yoshioka

**Affiliations:** 1 Biofunctional Imaging Laboratory, WPI Immunology Frontier Research Center (WPI IFReC), Osaka University, 3-1 Yamadaoka, Suita, Osaka 565-0871, Japan; 2 Functional Imaging Technology, Center for Information and Neural Networks (CiNet), National Institute of Information and Communications Technology (NICT) and Osaka University, Osaka, Japan; 3 Biotechnology Development Laboratories, Kaneka Corporation, Hyogo, Japan; 4 Department of Nephrology, Osaka University Graduate School of Medicine, Osaka, Japan; 5 Institute for Chemical Research, Kyoto University, Kyoto, Japan

**Keywords:** Super paramagnetic iron oxide, magnetic resonance imaging, kidney, mouse, stealth probe

## Abstract

**Introduction:**

To investigate the biodistribution and retention properties of the new super paramagnetic iron oxide (new SPIO: mean hydrodynamic diameter, 100 nm) nanoparticles, which have concentrated polymer brushes in the outer shell and are difficult for phagocytes to absorb, and to compare the new SPIO with clinically approved SPIO (Resovist: mean hydrodynamic diameter, 57 nm).

**Materials and Methods:**

16 male C57BL/6N mice were divided in two groups according to the administered SPIO (*n* = 8 for each group; intravenous injection does, 0.1 ml). *In vivo* magnetic resonance imaging (MRI) was performed before and one hour, one day, one week and four weeks after SPIO administration by two dimensional-the fast low angle shot (2D-FLASH) sequence at 11.7T. *Ex vivo* high-resolution images of fixed organs were also obtained by (2D-FLASH). After the *ex vivo* MRI, organs were sectioned and evaluated histologically to confirm the biodistribution of each particle precisely.

**Results:**

The new SPIO was taken up in small amounts by liver Kupffer cells and showed a unique *in vivo* MRI contrast pattern in the kidneys, where the signal intensity decreased substantially in the boundaries between cortex and outer medulla and between outer and inner medulla. We found many round dark spots in the cortex by *ex vivo* MRI in both groups. Resovist could be detected almost in the cortex. The shapes of the dark spots were similar to those observed in the new SPIO group. Transmission electron microscopy revealed that Resovist and the new SPIO accumulated in different cells of glomeruli, that is, endothelial and mesangial cells, respectively.

**Conclusion:**

The new SPIO was taken up in small amounts by liver tissue and showed a unique MRI contrast pattern in the kidney. The SPIO were found in the mesangial cells of renal corpuscles. Our results indicate that the new SPIO may be potentially be used as a new contrast agent for evaluation of kidney function as well as immunune function.

## Introduction

Super paramagnetic iron oxide (SPIO) nanoparticles have received attention in bioscience research since the first report in the 1980s.^1^ Because of the nonspecific uptake of SPIO by the mononuclear phagocyte system (MPS) and reticuloendothelial system (RES) after the administration, SPIO has been used in preclinical and clinical diagnostic magnetic resonance imaging (MRI) of organs and tissues, particularly liver, spleen, lymph nodes and bone marrow.^2–4^ The cellular uptake of SPIO also allows it to be used as a special contrast agent for labeling phagocytes. It enables to visualize phagocytes recruited into inflammatory lesions and to track their dynamic migrations by MRI,^5–7^ which plays an important role in the pathologic research nowadays. Many previous studies have aimed to improve the unique physicochemical and biological properties of SPIO by modifying particle structure, size and coating.^8,9^ In order to improve the properties for the applications such as drug targeting and tissue or organ imaging other than liver, it is extremely important to avoid the nonspecific uptake of SPIO by peripheral macrophages as well as by MPS and RES, which could increase the half-life of the particles in the blood.^10,11^ Ohno et al. reported that their synthetic technique renders the ferric oxide particle stealthy: only very limited amounts are absorbed by phagocytes.^12^ They fabricated hybrid particles (new SPIO) composed of a core of iron oxide magnetite (Fe_3_O_4_) nanoparticle and a shell of concentrated hydrophilic polymer brushes synthesized by surface-initiated living radical polymerization techniques. The new SPIO nanoparticles do not contain ‘silica’. The polymer brushes are composed by hydrophilic polymers, poly (poly [ethylene glycol] methyl ether methacrylate [PEGMA]).^12,13^ This new probe showed excellent dispersibility in aqueous media and a marked increase in blood circulation time due to its stealth characteristics. It is also considered that the new SPIO with specific ligands for various diseases could be used as a selective contrast agent of MRI.^11,14^ However, it is necessary first to assess the biodistribution, biocompatibility, and clearance of the new SPIO.^10^ Therefore, we aimed to investigate the biodistribution and retention property of this new SPIO in normal mouse body and to compare it with a clinically approved SPIO (Resovist; I’rom Pharmaceutical Co., Ltd.).

## Materials and Methods

### Animals and experimental models

16 male C57BL/6N mice aged 8 weeks were purchased from Japan SLC Inc. (Shizuoka, Japan). The mice were divided in two groups according to the SPIO they were administered (Resovist group: *n* = 8, new SPIO group: *n* = 8, intravenous injection does = 0.1 ml). The animals were housed under standard laboratory conditions (a 12-hour light/dark cycle, standard laboratory chow and water ad libitum). All animal experiment procedures in this study were approved by the Animal Research Ethics Committee of Osaka University.

### SPIO for MRI

We used two types of SPIO in this study.

- a commercially available SPIO, Resovist (I’rom Pharmaceutical Co., Ltd., Tokyo, Japan). Resovist is a dispersion of SPIO nanoparticles coated with carboxydextran^15^ and approved for clinical use. The mean hydrodynamic diameter of this SPIO is 57 nm, and the iron concentration is 28 mg/ml.
- new SPIO; stealth magnetic particles with concentrated polymer brushes that contain a little fluorophore, rhodamine. The particles were originally synthesized by surface-initiated living radical polymerization techniques,^13^ which are not almost taken by phagocytes due to the suppression of non-specific protein binding caused by their “brush-afforded” structure. The mean hydrodynamic diameter of this particle is 100 nm, and the iron concentration is 4.5 mg/ml.

To compare the contrast effect and biodistribution of Resovist with those of the new SPIO, we intravenously administered these two SPIOs to mice separately. Due to their super paramagnetic properties, they have a dominant effect on shortening T_2_ and ${\text{T}}_{2}^{*}$ and create low-intensity regions on ${\text{T}}_{2}^{*}$-weighted images. We evaluated the contrast effect and biodistribution of the particles by a sequential ${\text{T}}_{2}^{*}$ MRI.

### Experimental procedure

Following the induction of 1.2% isoflurane inhalational anesthesia, 2D-FLASH MRI of the abdomen of each mouse was obtained *in vivo* as the baseline image. After that, the suspension of the new SPIO was injected into the tail vein of mice at a dose of 200 μmol Fe/kg body weight using a 30-G needle (new SPIO group). Resovist was also injected for the comparison at a dose of 2 mmol Fe/kg body weight (Resovist group). At one hour, one day, one week and four weeks after particle administration, we performed *in vivo* MRI under 1.2% isoflurane inhalational anesthesia. After *in vivo* MRI scan of post four weeks, mice were sacrificed, and liver, spleen and kidney were excised after perfusion with phosphate buffered saline and fixation with 4% paraformaldehyde (*n* = 4 for each group). Then high-resolution images of fixed organs were obtained by 2D-FLASH sequence (*ex vivo* MRI). In order to get a better contrast of the image, fixed organs were soaked in the dilute gadolinium solution (5 mM) overnight before the scanning. After *ex vivo* MRI, organs were sectioned and evaluated histologically.

### In vivo MRI measurement

Following the induction of anesthesia as previously described, all the *in vivo* MRI was conducted using an 11.7T vertical-bore scanner (AVANCE II 500WB; Bruker BioSpin, Ettlingen, Germany) and a 25 mm inner diameter transmit/receive volume radio frequency (RF) coil. Coronal abdominal images of each mouse before and after SPIO administration were obtained using the fast low-angle shot (FLASH) sequence ( ${\text{T}}_{2}^{*}$ WI; repetition time [TR] = 400 ms, echo time [TE] = 3 ms, flip angle [FA] = 30°, number of averages [NA] = 8, field of view [FOV] = 25.6 mm × 25.6 mm, matrix size = 256 × 256, slice thickness = 500 μm, acquisition time = 13 min).

### Ex vivo MRI measurement

The *ex vivo* MRI scanning of excised kidney and liver soaked in dilute gadolinium solution (*n* = 4 for each group) were performed using a 10 mm inner diameter volume coil by the above 11.7T vertical scanner. The FLASH sequence was used with the following parameters: TR / TE = 500 ms / 6 ms, FA = 30°, NA = 128, FOV = 10 mm × 10 mm, matrix = 512 × 512, thickness = 0.12 mm, acquisition time = 4.5 hrs.

### Histology

After the *ex vivo* MRI, tissues were embedded in paraffin wax (*n* = 2 for each group) and sliced with a microtome at a thickness of 5 μm. Tissue sections were dewaxed in xylene and rehydrated by a series of ethanol-water mixtures. For the Resovist group, the Prussian blue staining and nuclear fast red counter staining were performed to verify the existence of particles in each tissue. Microscopic images were captured from stained 5-μm paraffin-embedded sections using an FS × 100 microscope (Olympus, Tokyo, Japan). The new SPIO, however, cannot be detected by the Prussian blue staining due to the special surface modification. Therefore, we observed the rhodamine fluorescence to verify the existence of particles in each tissue of the new SPIO group. As described previously for the Resovist group, tissues of the new SPIO group were also embedded in paraffin wax and cut at a thickness of 5 μm. After the deparaffinization, antigen was retrieved by incubating the sections in 10 mM citrate retrieval buffer solution at 120^°^C for 10 min. To block endogenous peroxidase activity, sections were incubated in peroxidase-blocking solution (3% H_2_O_2_ in methanol) for 20 min and rinsed with distilled water, followed by a 5-min rinse in phosphate buffered saline (PBS). Sections were blocked in a solution of 10% fetal bovine serum (FBS) with 1% bovine serum albumin (BSA; Sigma Chemical, St. Louis, MO, USA) for 1 h at room temperature. The sections were then washed three times in PBS for 5 min each and mounted with ProLongH Gold Antifade Reagent with DAPI (Invitrogen). Microscopic images of stained sections were captured using an FS × 100 microscope.

### Transmission electron microscopy

After the *in vivo* MRI of post 1 week, mice (*n* = 3 for each group) were sacrificed, and their kidneys were harvested after the perfusion with heparinized PBS and the fixation with 4% (w/v) paraformaldehyde in 0.1 M phosphate buffer solution (pH 7.4). The kidneys were cut in small cubes (roughly 0.5-mm sides) as samples for transmission electron microscopy (TEM). The cut tissue samples were washed three times for 5 min each in 0.1 M phosphate buffer solution (pH 7.4) containing 4% sucrose. The samples were then post fixed by 1% (w/v) OsO_4_ and 1% (w/v) potassium ferrocyanide in 0.1 M phosphate buffer solution (pH 7.4) for one hour at room temperature in a draft chamber, and washed three times for one min in PBS. All the samples were dehydrated in a graded series of ethanol solutions: 50%, 70%, and 90% (v/v) ethanol on ice, then twice in 100% ethanol for 10 min at room temperature. Then the sample tissues were incubated in 50% (v/v) epoxy resin mixture dissolved in 100% ethanol for 60 min at room temperature. The dehydrated tissues were infiltrated with resin mixture for one hour at room temperature. This process was then repeated. The embedding mixtures were polymerized in the cavities of silicon rubber for 2 days at 60^°^C. Marked regions of the resulting resin blocks were cut into ultra-thin sections of 60–90 nm. The thin sections were stained with 2% (w/v) uranyl acetate solution for one hour and briefly washed three times in distilled water. The sections then were incubated in lead-staining solution for two min and washed briefly three times in distilled water, then scanned by an electron microscope at 80 kV (JEM-1011; JEOL, Tokyo, Japan).

## Results

**Fig 1.**
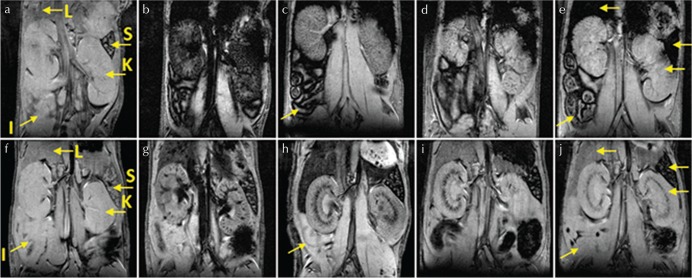
Mouse abdomen magnetic resonance imaging (MRI) before and after the intravenous administration of Resovist (**a**–**e**) and the New super paramagnetic iron oxide (SPIO) (**f**–**j**). Representative slices of mouse abdomen are shown at each time point: before (**a**, **f**), 1 hour post (**b**, **g**), 1 day post (**c**, **h**), 1 week post (**d**, **i**), 4 weeks post (**e**, **j**) the administration of these two particles. Characters L, S, K and I indicate liver, spleen, kidney and intestines, respectively.

**Fig 2.**
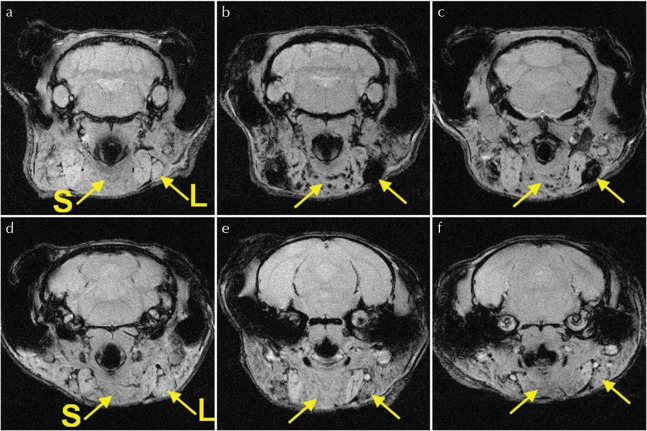
Mouse head magnetic resonance imaging (MRI) before and after the intravenous administration of Resovist (**a**–**c**) and the New super paramagnetic iron oxide (SPIO) (**d**–**f**). MRI shows salivary glands and cervical lymph nodes at each time point: before (**a**, **d**), 1 week post (**b**, **e**), 4 weeks post (**c**, **f**) the administration. Characters S and L indicate salivary glands and lymph nodes, respectively.

**Fig 3.**
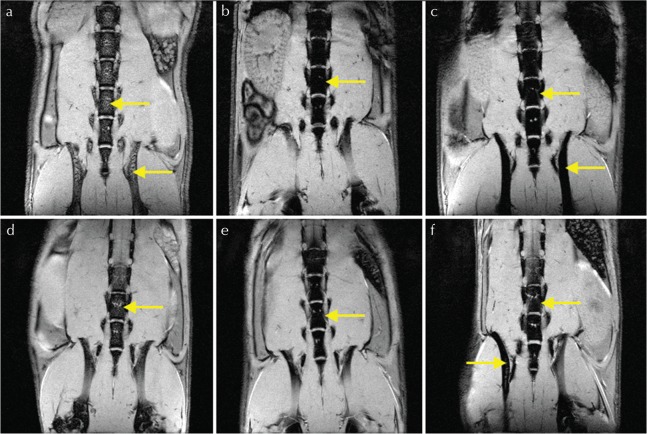
Mouse bone marrow magnetic resonance imaging (MRI) before and after the intravenous administration of Resovist (**a**–**c**) and the New super paramagnetic iron oxide (SPIO) (**d**–**f**). MRI shows trabecular and iliac bones at each time point: before (**a**, **d**), 1 week post (**b**, **e**), 4 weeks post (**c**, **f**) the administration. Yellow arrows indicate bone marrows (trabecular and iliac bones).

**Fig 4.**
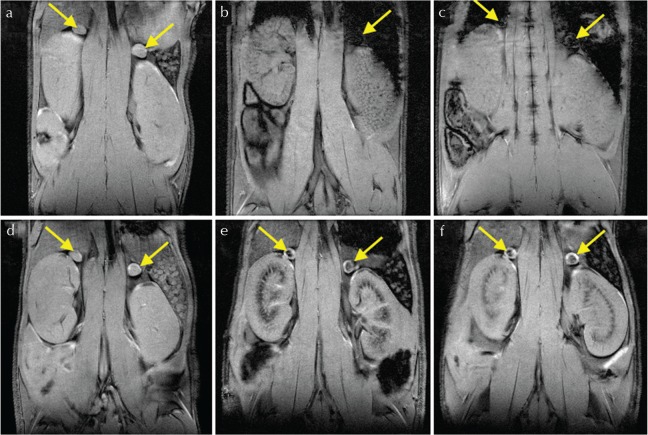
Magnetic resonance imaging (MRI) of adrenal glands before and after the intravenous administration of Resovist (**a**–**c**) and the New super paramagnetic iron oxide (SPIO) (**d**–**f**). MRIs were obtained at each time point: before (**a**, **d**), 1 week post (**b**, **e**), 4 weeks post (**c**, **f**) the administration. Yellow arrows indicate adrenal glands.

The abdomen *in vivo* MRI of Resovist group (Fig. 1a–e) showed a marked decrease of signal intensity in blood vessels, liver and spleen just after injection. The signal intensity of blood vessels recovered to the level before injection within a day. Resovist, however, retained for a long time in the liver, spleen and intestines (Fig. 1d, e). The signal intensities of lymph nodes and bone marrow were as low as those of the liver and spleen, for a long time (Figs. 2c and 3c). Salivary glands showed several small dark regions even at four weeks (Fig. 3a–c). On the other hand, the new SPIO showed the transient decrease of signal intensities in blood vessels and lymph nodes (Figs. 1f–j, 2d–f). The signal intensity in the liver had almost recovered at one week after injection (Fig. 1i), but the intensities in spleen and bone marrow remained low even at four weeks (Figs. 1j, 3f). The new SPIO showed a slight decrease in the signal intensity in intestines (Fig. 1h–j). Salivary glands of the new SPIO did not show the decrease of the intensity (Fig. 2d–f). Both SPIOs showed a low signal intensity region in adrenal grands even at 4 weeks (Fig. 4). Unexpectedly, the new SPIO showed a unique contrast pattern in live mice kidneys (Fig. 1h–j) which was quite different from that of Resovist group (Fig. 1c–e). The new SPIO produced regions of low signal intensity in the boundaries between cortex and outer medulla and between outer and inner medulla, and the recovery speed of the low-signal regions was slow (Fig. 1j). The Resovist group, on the other hand, exhibited a small decrease in signal intensity in the kidney cortex at one and four weeks after injection (Fig. 1d, e).

**Fig 5.**
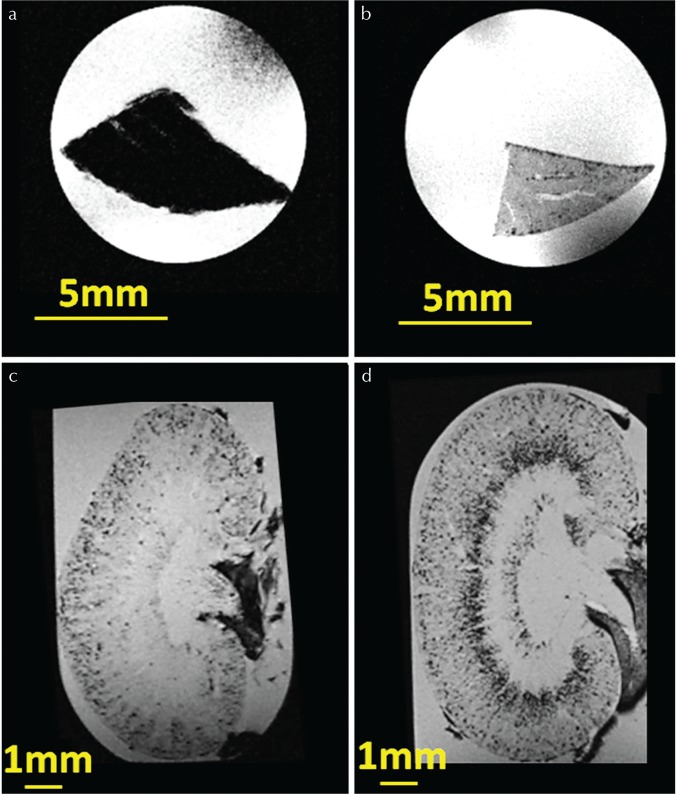
*Ex vivo* high-resolution magnetic resonance imaging (MRI) of liver and kidney at 4 weeks after the administration of Resovist or new super paramagnetic iron oxide (SPIO) (*n* = 4 each). (**a**), liver of Resovist group. (**b**), liver of new SPIO group. (**c**), kidney of Resovist group. (**d**), kidney of new SPIO group.

To examine the more detailed biodistribution of particles in the liver and kidneys of each group at four weeks after SPIO injection, we carried out *ex vivo* MRI scans after perfusion fixation. Liver samples from the Resovist group were dark even at 4 weeks after SPIO administration (Fig. 5a). On the other hand, we could only observe a few dark spots in the liver of new SPIO group (Fig. 5b). The Resovist group exhibited dark round spots in the kidney cortex (Fig. 5c), which looked like renal corpuscles. We confirmed that the dark spots by the new SPIO were found much in the boundaries between cortex and outer medulla and between outer and inner medulla (Fig. 5d). The number of dark spots was higher in the cortex than in the medulla. The distributions and shapes of dark spots found in the cortex of Resovist and the new SPIO groups were quite similar.

**Fig 6.**
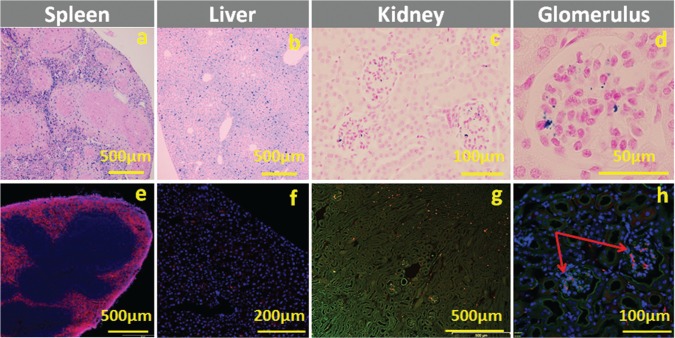
Histological images of mouse tissues at 4 weeks after the administration of Resovist or new super paramagnetic iron oxide (SPIO) (containing a little fluorophore, rhodamine). The upper row shows Prussian blue staining of the Resovist group. The lower row shows fluorescence images of the new SPIO group. Red arrows (**h**) indicate glomeruli.

**Fig 7.**
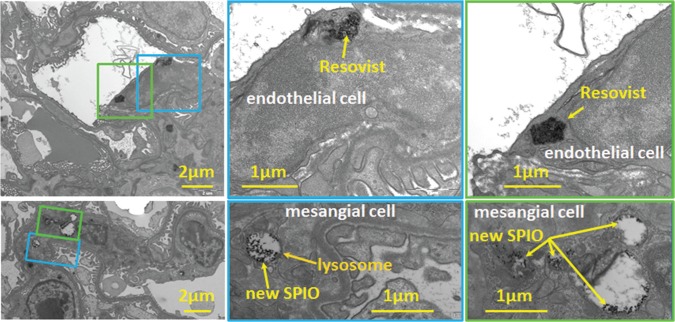
Transmission electro-microscope images of kidneys at 1 week after the injection of Resovist (upper row) or New super paramagnetic iron oxide (SPIO) (lower row).

The biodistribution of particles in tissues and cells was also examined histologically after the *ex vivo* MRI. We observed a large number of particles deposited in the liver and spleen of Resovist group (Fig. 6a, b). In the kidney, Resovist was found in glomeruli (Fig. 6c, d). These results were consistent with that we found by *ex vivo* MRI. We found a large number of new SPIO in the red pulp of the spleen but rarely in the liver (Fig. 6e, f). In the kidney, we detected many new SPIO in glomeruli and in the boundaries between cortex and outer medulla (Fig. 6g, h), which was also consistent with the MRI results. The TEM images of kidney tissues showed that the most Resovist was engulfed by the endothelial cells of the glomeruli (Fig. 7a–c). On the other hand, most of the new SPIO were found in the mesangial cells of the glomeruli (Fig. 7d–f).

## Discussion

In this study, we investigated the biodistribution and retention properties of the new SPIO in normal mice, for comparison with a clinically approved SPIO (Resovist) using ultra-high magnetic field *in vivo* and *ex vivo* MRI. The sequential MRI tracking of these two particles also allowed us to detect the difference of pharmacokinetics between them. We observed that Resovist showed a persistent decrease of signal intensity in liver, lymph node, spleen and bone marrow (Figs. 1–3) as expected. This indicates the extensive duration of Resovist accumulation in these organs and tissues.^2–4^ This may be caused by the nonspecific uptake of Resovist in the MPS and RES of these tissues after the administration.^16^ Resovist showed the decrease of signal intensities in the intestines and salivary glands (Figs. 1, 2). On the other hand, the new SPIO showed transient signal reductions in these organs. These results indicate that the signal reductions in these organs by Resovist were caused by phagocytes,^17,18^ where the antigen presentation by phagocytes is important. The labeling of phagocytes in the intestines and salivary glands by Resovist would be useful to assess the immunological responses in these organs.^17–19^ We found the accumulation of Resovist and the New SPIO in adrenal grand as well as bone marrow and spleen even at 4 weeks (Fig. 4). Hume et al. showed the existence of macrophages in the adrenal cortex and medulla.^20^ However, the mechanism of the accumulation of Resovist and the new SPIO should be different because the new SPIO is taken little by macrophages. MRI of adrenal glands as well as bone marrow and spleen with Resovist and the new SPIO would provide different information about the function of these organs.^21,22^

Resovist has been used clinically because of its high efficiency for the detection and characterization of small focal liver lesions.^23^ The problem of the particle is the propensity of clearance in liver, spleen, bone and lymph nodes, although the particle is good for the imaging of macrophage rich organs and tissues.^24^ This tendency decreases the tool’s specificity and limits the applications in the context of clinical diagnosis.^11^ There are so many studies on the improvement of the specificity of SPIO to expand their use in biomedical applications.^9,10^ The new SPIO we used in this study showed a transient decrease of signal intensity in the liver and blood vessels, and we found no accumulation of the new SPIO in lymphatic tissues (Figs. 1, 2). This may be because of the special coating structure (brush afforded), which make the particle stealthy and difficult for phagocytes to absorb.^12,13^ Due to its stealth, the new SPIO may provide a specific functional contrast agent by conjugating with other biomarkers for MRI in the future.^25^ The new SPIO may also be a good candidate of imaging nanoparticles for theranoustics reagents.^11^ Its high clearance rate in liver tissue also suggests the possibility of repeated usage in the same subject.

Our study also revealed that the new SPIO showed a unique distribution pattern in mouse, especially in kidney (Figs. 1, 5), which was not observed with other contrast agents. This result suggests us the possibility to apply this new SPIO for a diagnostic tool of kidney inflammation, because the distribution of the new SPIO may change when the inflammation occurs in the kidney.^26^ Previous studies showed us that changes in the number and size of kidney glomeruli have been linked to various renal and systemic diseases.^27,28^ It has a clinical significance to estimate the number of glomeruli *in vivo*. Beeman’s group showed the possibility to measure glomerular number and size of kidney by the injection of cationic ferritin with ultra-high magnetic field MRI.^29–31^ Our MRI shows the unique accumulation of new SPIO in the glomeruli and indicated that the number of glomeruli could be estimated by the administration of the new SPIO (Figs. 5, 6). MRI with the new SPIO could be used for longitudinal studies of focal and segmental renal diseases and could monitor the progress of therapy in the future as same as the cationic ferritin, though the accumulation mechanism of cationic ferritin and the new SPIO may be different.^32^

The TEM images of kidney glomeruli showed that Resovist and the new SPIO were engulfed by the endothelial and mesangial cells of the glomeruli, respectively (Fig. 7). This indicates that Resovist and the new SPIO have an entirely different mechanism of accumulation in kidney glomeruli. To the best of our knowledge, the image provided here showing the uptake of Resovist and the new SPIO by glomerular cells is the first of its kind.

Even though the new SPIO was absorbed in only negligible amounts by phagocytes in liver and lymphatic tissues due to its stealth, numerous particles were engulfed by mesangial cells in kidney glomeruli. Mesangial cells could recognize particles which macrophages could not recognize. This indicates that the immune mechanism of kidney might be different from that of other tissues. As the mesangial cells occupy a central anatomical position in glomeruli and also play an important regulatory role in immune mediated glomerular diseases, it would be of great clinical significance if we can evaluate the function of mesangial cell by an *in vivo* method.^31^ The unique distribution of the new SPIO in normal mesangial cells could be used as a marker to assess the glomerular and immune functions of kidney. Our results indicate that MRI using the new SPIO may reflect tissue functions, inflammations, and diseases of the kidney, and that this new SPIO may be potentially be used as a new diagnostic tool for nephritis in the future.

## Conclusion

We found that the new SPIO is taken up in only negligible amounts by mouse liver. On the other hand, mouse kidney exhibited a unique MRI contrast pattern after receiving the new SPIO. This SPIO was found in the mesangial cells of renal glomeruli by TEM. Our results indicate that the new SPIO may be potentially be used as a new contrast agent for the assessment of kidney function because mesangial cells play important roles in renal physiology and immune function.
